# The effect of audit team’s emotional intelligence on reduced audit quality behavior in audit firms: Considering the mediating effect of team trust and the moderating effect of knowledge sharing

**DOI:** 10.3389/fpsyg.2022.1082889

**Published:** 2022-12-13

**Authors:** Mingyuan Zhao, Yuyue Li, Jie Lu

**Affiliations:** Pan-Asia Business School, Yunnan Normal University, Kunming, China

**Keywords:** audit team, emotional intelligence, team trust, knowledge sharing, reduced audit quality behavior, social exchange perspective

## Abstract

Reduced audit quality behavior is widespread in the auditor’s practice and is an important factor threatening audit quality. Some prior studies have investigated the relationship between auditors’ psychological contract violation and reduced audit quality behavior. However, the research of relationship between emotional intelligence (EI) and auditors’ behavior is still in its infancy despite the fact that the auditing profession would benefit greatly from improving audit team’s EI. This study examines whether and why the audit team’s EI restrains the audit quality reduction behavior in audit firms. In the study, our hypotheses are tested using a data set collected from 326 respondents in Chinese audit firms. The results are as follows: firstly, audit team’s EI is directly negatively related to reduced audit quality behavior. Secondly, EI is indirectly related to reduced audit quality behavior, through team trust. The results of structural equation modeling (SEM) indicate a mediation model where team trust is negatively related to reduced audit quality behavior. Thirdly, knowledge sharing is a significant mechanism that moderates the effects of different types of EI on audit quality reduction behavior. In the audit team with high knowledge sharing, the audit team’s EI can refrain the audit quality reduction behavior; In the audit team with low knowledge sharing, the audit team’s EI has no significant effect on audit quality reduction behavior. This study expands the factors affecting audit quality to the psychological level of audit teams, enriches the literature on audit team’s behavior characteristics, and provides direct evidence for the relationship between audit team’s psychological characteristics and audit quality.

## Introduction

This study examines whether and why the audit team’s emotional intelligence (EI) restrains the audit quality reduction behavior in audit firms. Emotional intelligence refers to the ability of individuals to perceive, express, and manage their own emotions, recognize the emotions of others, and use this information to guide their own thinking and actions (Joseph and Newman, 2010). When high-level interpersonal interaction and emotional clues appear in the audit team, the impact of individual EI can be reflected to the team level through the activated interpersonal communication mechanism, and the aggregation of individual EI will form a group EI at the team level. The EI of an audit team is the ability of an audit team to develop a set of norms for managing emotional processes. These norms encourage the expression and regulation of emotional dynamics within and outside the team, thus helping team members to deal with emotional problems more effectively (Curseu et al., 2015).

Auditing is naturally a team-based attribute. This is because in terms of cost inputs for an audit firm, no matter how much emphasis is placed on the importance of IT, management coordination, logistics, or other related inputs for the audit firm as a whole, labor costs have always been and will remain a core part of audit costs in the future. When doing a particular audit project, instead of devoting all of the firm’s human resources to a single client, only one audit team needs to be dispatched. Most of the audit work is carried out in an audit team organization. To better manage audit teams and achieve results, recent studies have highlighted the importance of EI for teams to achieve performance (Montenegro et al., 2021; Zhu et al., 2021). Audit teams consist of individual auditors with a variety of skills. Individual auditors influence the level of audit quality, but auditors do not work in isolation; they are influenced by the EI of the audit team they work with. It has been argued that firms that effectively manage emotions within their organizations deliver better performance and higher returns than firms that ignore them (Parmar et al., 2010). Audit quality is the most central performance indicator of the audit team and is one of the most important topics in the audit profession. However, reduced audit quality behavior is widespread in auditors’ practice and is an important factor threatening audit quality. Reduced audit quality behavior is defined as actions taken by an auditor during an engagement that reduce evidence-gathering effectiveness inappropriately (Herrbach, 2001). The behaviors related to the reduction of audit quality are of concern to audit firms and industry regulators, because they seem to be systematic. Previous studies have shown that more than half of the auditors admit to participating in at least one of the behaviors of reducing audit quality (Coram et al., 2003). In prior studies, the main variables for reduced audit quality are as follows: auditor firms’ quality control and review procedures (Aobdia, 2020), time budget pressure (Coram et al., 2004), auditors’ personality type (Gundry and Liyanarachchi, 2007), auditor independence and competence (Dart and Chandler, 2013), and high workloads (Persellin et al., 2019). The quality of the audit process is considered high if the auditor is able to detect and report existing material misstatements (Mohamed and Habib, 2013).

To restrain the behavior of auditors’ quality reduction depends not only on the competence or competency of audit team members, but also on being able to manage their emotional intelligence. According to view of Goleman (2001), the more complex the job, the more important emotional intelligence becomes, and emotional intelligence affects a person’s behavior from within. People with high EI are able to make informed decisions, cope better with environmental demands and pressures, handle conflict effectively, communicate in an exciting and assertive manner, and make others feel better in their work environment (Love et al., 2011). Although the effects of team project leaders (Lennox et al., 2018), auditor overconfidence (auditor overconfidence), auditor narcissism (Khaksar et al., 2021), auditor resilience (Smith and Emerson, 2017), and auditors’ psychological contract violation (Herrbach, 2001) on audit behavior have been explored in EI management research, the relationship between the audit steam’s EI and reduced audit quality behavior has not been described. Therefore, the first key research question of this study is as follows.

*Q1.* What is the relationship between the audit team’s EI and reduced audit quality behavior?

In addition, the relationship between audit team’s EI and reduced audit quality behavior may be complex and cannot be explained simply by direct effects. EI is a group of abilities to process emotional information, and its effectiveness depends on the degree of effective recognition and utilization of emotional information (Fineman, 2006). EI is consciously released during interactions with team members to drive and screen the responses of others. In this process, EI can not only be publicly displayed but also identify emotions from others (Ashkanasy and Dorris, 2017). People with high EI are more likely choose emotional strategies, such as eliciting, faking, promoting, and inhibiting emotions, to display their EI to team members by adopting the best strategy (Humphrey, 2013). EI is usually positively correlated with extraversion and negatively correlated with neuroticism, while it also shows a small significant positive correlation with openness, agreeableness, and conscientiousness (Saklofske et al., 2003). Based on the research base of this literature, this study aims to provide evidence on how audit teams’ EI affects reduced audit quality behavior, and address the second question:

*Q2.* What is the mechanism of audit team’s EI impacts on reduced audit quality behavior?

In order to solve these two problems, based on the literature on the social exchange theory (Obrenovic et al., 2020) and the theory of planned behavior (Ajzen, 1991), this study established a theoretical model related to the five hypotheses. According to social exchange theory, social behavior involves a social exchange in which individuals are motivated to obtain a reward, for which they must give up something valuable (Obrenovic et al., 2020). Therefore, reduced audit quality behavior is a result influenced by the teams’ EI. Auditors are willing to refrain the audit quality reduction behavior and according to expectancy theory, they aim to gain monetary or non-monetary rewards such as bonuses, promotions, and trust. Central here is the concept of reciprocity. Theory of planned behavior suggests that attitudes and subjective norms can be used to explain audit quality reducing behaviors. Individuals are more willing to comply with norms when they believe that the team’s EI facilitates behaviors that improve audit quality. Trust among audit team members affects auditors’ attitudes toward their audit engagements.

The five hypotheses were obtained through questionnaires. After reliability and validity tests, a structural equation model was constructed to test the theoretical model. The results show that the audit team’s EI can restrain the behavior of auditors’ quality reduction. After data analysis, we propose a specific mechanisms of how high EI individuals use emotional information to influence others’ knowledge. The mechanism involves the process of influencing oneself. Individuals with high EI are good at using emotional clues to change their emotions, are willing to share their knowledge with team members, and promote the professional competence of team members through knowledge sharing to ensure audit quality. The mechanism is also to consciously release emotional information in social interaction, stimulate other’s emotion of knowledge sharing, or dynamically evaluate emotional state at any time, and then take beneficial strategies to achieve the desired goal.

Our theoretical views will make significant contributions to research on reduced audit quality behavior: Firstly, this study expands the research on emotional intelligence in audit behavior by introducing the research on inhibition and reduction of audit behavior by emotional intelligence of audit teams, and contributed to the literature on emotional psychology. Secondly, this paper reveals that in audit teams with high audit EI, knowledge sharing is conducive to inhibiting the behavior of reducing audit quality, and contributes to the literature of knowledge sharing. Thirdly, this study expands the channel research of EI on project results. The prior research of channel mostly focuses on positive behavior, on work context and on leadership types (Cavazotte et al., 2012). Our empirical results show that team trust is the channel through which audit team’s EI affects audit quality.

The following section reviews relevant literature regarding the theory of EI intelligence and audit quality reduction behavior, and proposes five hypotheses among key constructs; subsequently, a methodology for collecting 326 usable questionnaires, measuring constructs, and testing for reliability and validity are displayed. Then, the regression model of data is provided. Finally, the study’s findings, discussion, theoretical implications, managerial implications, and limitations are presented.

## Literature review and hypotheses

### Audit team’s EI and team trust

There are two views on EI, one is the ability EI, and the other considers EI in a more mixed perspective. This study is based on the theory of ability EI, and draws on the existing calculation methods. When calculating the audit team’s EI score, the audit team’s EI is realized by the accumulation of individual EI, that is, first measure the individual EI, then add them up and average them.

Team trust is a positive expectation of traits, such as competence, sincerity, integrity, and reliability of others in the team. Managers with high EI are able to demonstrate their vision more persuasively to their employees and manage social networks effectively, which not only helps them to build and maintain trust with stakeholders, but also to access information and resources (Naude et al., 2014). Team EI can improve team satisfaction, trust levels, and increase team cohesion, among others (Chang et al., 2012). When team EI is low, teams often adopt a negative approach to deal with problems, which has a negative impact on team trust, etc.; when team EI is high, teams are more likely to adopt a cooperative approach to deal with problems and resolve conflicts, which has a positive impact on team trust. In audit teams, team EI still plays a similar role. The relationship between the two dimensions of team EI on team trust is discussed separately below:

#### Analysis at individual level

There is a positive relationship between individual dimensions of EI and trust (Chun et al., 2010). Individuals with high EI are more likely to perceive intra-team trust. The logic is that, firstly, people with high EI can effectively identify trustworthy behaviors in a given context. During social interactions, most people want to present themselves in a trustworthy manner and want this behavior to be perceived by others. People with high EI have stronger social interaction and relationship management skills, and the above skills and abilities help them make correct attributions about the motivations and behaviors of team members, thus creating a stronger sense of trust in the team.

Secondly, in dealing with negative events, people with low EI usually exhibit distrustful behaviors, while the opposite is true for people with high EI. In the process of teamwork, people with low EI usually feel helpless when faced with others’ misbehavior such as not completing work tasks on time or not meeting work requirements, and give up the opportunity to further seek the real cause of the misbehavior, and often attribute the misbehavior to lack of ability or intentional behavior, and this attribution tends to make them think that the other party is not trustworthy. When faced with the same situation, people with high EI are more likely to trust others, and they tend to attribute the misconduct to uncontrollable factors (e.g., lack of professional competence), and to learn the true cause through further communication or other means, while thinking more about how to improve the situation and taking the right actions (e.g., sharing audit experience, giving moral motivation, etc.) to remedy it. This trust makes it easier to develop trust in the team.

Thirdly, people with high EI are more likely to experience positive emotions than those with low EI (Fredrickson, 2001). Positive emotions expand the ability of team members to think and act instantaneously. When audit team members are in a positive emotional state, they are more open to information, more flexible and integrated in their thinking, and more likely to find positive meaning in events and generate more positive evaluations.

#### Analysis at team level

Social network theory asserts that in the context of a team project, team members must divide the work among themselves and communicate adequately during the project in order to complete the task within a given time frame. EI influences the selection of others as friendly partners through the perception of team trust. When one chooses others as friendly partners, this positive expectation of the overall team influences one to have positive emotional experiences with team members, which in turn motivates more interactions and results in others choosing oneself as a friendly partner, thus effectively building team identity and team trust (Sparrowe et al., 2001). Teams with higher levels of EI usually have frequent communication, cooperation, and mutual understanding among their members and are prone to trusting cognitive behaviors. Therefore, this study proposes the following hypothesis:

*H1:* In the audit team, EI has a positive impact on team trust.

### Audit team’s EI and reduced audit quality behavior

From the point of view of audit practice, audit work should be “people-oriented,” but also “team work.” The individual auditor is an important determinant of project quality, and individual behavior is necessarily dependent on the work team, and the individual interests of the auditor are integrated with the interests of the team. At the same time, because auditors interact with client management extensively during the audit process, auditing is a job with strong EI implications. More generally, the reputation of the audit firm and the reasonableness of its charge level require that it have a high integrity image. In the long run, the audit firm cannot have negligent employees. In this sense, the reduced audit quality behavior must still be the concern of audit firms, especially in the case of continuous evolution of audit methods. In this case, the individual auditor has more freedom. A more judgmental approach leaves more room for auditor initiatives. This is positive to some extent, but the new approach also depends more on the conscience of individual auditors. The relationship between the two levels of team EI on reduced audit quality behavior is discussed separately below:

#### Analysis at individual level

Firstly, personal EI can help audit team members improve their interaction skills with others and exchange audit experience in the industry. The perception and understanding of industry experience are conducive to the audit team to cope with changes in environment. The auditor’s personal industry experience reflects the auditor’s professional competence, specifically, the auditor has accumulated and mastered the business characteristics, transaction processes, and special accounting policies of the customer’s industry. Auditors with high EI can help employees perceive problems from multiple perspectives, thereby improving employees’ self-awareness and skills (Sheldon et al., 2014). This helps other auditors make reasonable audit judgments and propose effective audit implementation plans, thus improving audit quality. In collaborative teams, communication and coordination mechanisms appear to be more important than control and command relationships (Chin et al., 2022). In addition, EI enables individuals to have a keen understanding of the dynamics of interpersonal relationships. It enables individuals to adjust their emotions more quickly according to the environment, which helps to strengthen personal interpersonal skills and improve social and political skills (Zaccaro et al., 2018).

Secondly, individual EI can promote auditors’ proactive behavior. Due to effective emotion regulation, members with high EI are more likely to show positive behaviors than those with low EI (Kim et al., 2005). This motivates auditors to use their specific industry expertise to gain a deeper understanding of their clients’ operating characteristics, transaction processes, and the accounting policies customary in the industry to better identify the risks of their clients’ financial reports and more accurately assess the fairness of their clients’ financial report generation and disclosure. The auditor’s expertise prevents the risk of potential audit failures from threatening and damaging the team’s reputation, thereby improving audit quality. In addition, in order to develop social relations, individuals with high EI may also adopt positive behaviors related to emotions (such as using humor to manage conflicts), obtain higher performance through emotional motivation, and establish relationships with others through self-monitoring (Cheung and Tang, 2009). This development of social relations has promoted the formation of the audit team’s cooperation ability. Team cooperation ability is an important “soft power” support behind the high-quality audit services provided by the firm. If the audit team has no “cooperation,” it will be degraded to “self-employed.” In the audit team, teamwork is particularly important.

Thirdly, individual EI can promote auditors to obtain more excellent performance outcomes. More and more studies have proved the positive relationship between EI and performance outcomes (Deming, 2017), which include decision quality, task performance, and productivity. Specifically, individuals with high EI also affect others’ emotional state or behavior tendency through the exchange of social emotional resources, thus affecting others’ work performance (Vidyarthi et al., 2014). Auditors with high EI can well control their emotions, exchange information with customers in the process of interaction with them by virtue of the social skills brought by EI, obtain sufficient and appropriate audit evidence, provide reasonable basis for issuing audit opinions, and efficiently refrain the audit quality reduction behaviors.

#### Analysis at team level

Firstly, team EI facilitates the emotional climate of emotional expression and experience of the members of the formal audit team. Emotions are often considered to be drivers of behavior and ultimately affect employee performance (Ashkanasy et al., 2017). Project leaders with high EI can create or maintain a cohesiveness within the team by stimulating positive group identity, establishing group norms, or encouraging team members to engage in emotional expression (Wilderom et al., 2015). This cohesiveness can alleviate the auditor’s multiple pressures of work deadlines, performance appraisals, and liability risks, enhance professional discretion, and improve audit quality.

Secondly, team EI can facilitate conflict management in teams. Conflict is a reflection of the emotions in a team (Jordan and Troth, 2004). Because team leaders with high EI are able to more accurately understand their own internal emotions and needs, they can also develop workplace norms that are accepted by the group, thereby reducing the occurrence of team conflict and maintaining a harmonious atmosphere in the team (Wilderom et al., 2015). Conflict reduction facilitates the formation of emotional alliances and alliances of interest in the audit team to ensure that the expected audit quality is achieved.

Thirdly, team EI helps audit teams to make better decisions to improve audit efficiency. There is a positive relationship between group EI and team efficiency (Jamshed and Majeed, 2019). Groups with high EI create an emotional climate that enables members to perceive the information expected by the organization and generate the corresponding emotions or motivations. For example, an atmosphere of openness and cooperation promotes the emergence and proliferation of new ideas. In this case, emotional contagion among audit team members will help improve the review process of audit and refrain an act of audit quality reduction. Therefore, this study proposes the following hypothesis:

*H2:* In the audit team, EI has a negative impact on reduced audit quality behavior.

### Team trust and reduced audit quality behavior

Trust is the basis of all economic exchange. An environment with a high level of trust increases organizational efficiency (Williamson, 1993). In an audit team, the quality and effectiveness of each member’s work assignments are often not fully under his or her control, as each member’s work assignments are more related to the work of others. If members are to do their assigned work well, the more critical factor is to gain the trust of others and make other team members willing to work with them. If trust is lacking, the work of the audit team cannot be carried out properly and in a timely manner.

By perceiving trust in the team, team members develop perceived cohesion, integrate organizational goals with their personal goals, and are more willing to work creatively. Attitude, as an internal psychological tendency, affects audit results. Early in the establishment of virtual teams, 2/3 of high-performing teams exhibit rapid trust and are able to maintain and sustain trust throughout the team’s duration (Kanawattanachai and Yoo, 2002). Trust helps to enhance the psychological security of team members, reduce the risk of trust among team members, believe that it is relatively safe to take interpersonal risks within the team, encourage team members to propose innovative audit ideas and methods, give full play to each team member’s professional and collaborative abilities, and improve the efficiency of audit team collaboration. Trust helps team members to be consistent in their understanding of important audit judgments and audit conclusions, improves the efficiency of audit procedures, reduces or avoids inefficient work caused by mistrust and disagreement, and ensures that audit quality reduction behavior is minimized. Therefore, this study proposes the following hypothesis:

*H3:* In the audit team, team trust has a negative effect on reduced audit quality behavior.

### The mediating role of team trust

In the audit team, higher team EI can effectively establish emotional norms, so that team members can put aside their doubts and guesses as soon as possible, and establish team trust. This atmosphere is conducive to the sharing of professional knowledge and insight audit information among team members. Employees with high EI who share resources will gain the trust and respect of other employees in the organization, obtain emotional satisfaction, and establish a good reputation, which will in turn make them more motivated to perform high-quality audits (Yan et al., 2016).

In contrast, when the EI of the audit team is low, team members are in a relatively unfamiliar environment and are prone to emotional reactions such as suspicion and skepticism, resulting in mistrust among members and a failure to cooperate and share. A distrustful working atmosphere can cause auditors to behave in a dysfunctional manner, which in turn leads to an increase of audit quality reduction behaviors (Lopez and Peters, 2012). According to the basic principles of the theory of planned behavior, when planning behavior, alternative choices are analyzed to determine that the choice is most likely to achieve the desired goal. When auditors believe that working together makes the accomplishment of the target assignment more likely, they will be willing to regulate their audit behavior under conditions of trust in order to obtain the achievement of the team’s audit objectives. Therefore, this study proposes the following hypothesis:

*H4:* In an audit team, team trust plays a mediating role between audit team’s EI and reduced audit quality behavior.

### The moderating role of knowledge sharing

Audit firms as a knowledge-intensive organization, knowledge is more important component of core competency for their audit teams. Knowledge sharing is the basis for firms to acquire and sustain competitive advantage (Felin and Hesterly, 2007). To perform an efficient and effective audit, an auditor must have knowledge of several aspects, such as general domain knowledge of accounting and auditing standards, sub professional knowledge related to specific industries or customers, and general business knowledge. When working with alliance partners with heterogeneous knowledge, auditors can acquire humanistic and audit knowledge that reflects personal insights through mutual learning (Cohen and Caner, 2016; Chin et al., 2021). By the social exchange theory, the willingness to share knowledge is high in the expectation of reciprocal benefits.

More frequent communication between audit team leaders and members can also reduce the latter’s role conflict and ambiguity, thus promoting more, and more focused, proactive transmissions. Audit team members share knowledge with other members and diffuse knowledge from individuals to teams, which facilitates the construction of a firm’s competitive advantage. Knowledge sharing in audit teams is demonstrated by the fact that rapid team trust in the development process facilitates knowledge sharing among members, and good knowledge sharing also positively affects rapid trust (Chow et al., 2008). Knowledge sharing can improve audit quality by increasing the audit team’s ability to adequately use accumulated industry expertise to better identify and respond to risks of material misstatement of financial statements.

In teams with a high level of knowledge sharing, team members are willing to share their knowledge to other members. This creates a harmonious atmosphere and good member relationship for the team, improves the overall capability of the team members, and brings high quality audit opinions to the audit team. In contrast, in teams with low knowledge sharing, members are reluctant to share their knowledge, skills, etc. to other members. These team members keep knowledge confidential and guard against knowledge transmissions. This may make team members less inclined to seek advice from other members or to reveal adverse audit findings. These behaviors reduce the quality of the audit. In other words, knowledge sharing has an enhanced effect on audit quality. Previous studies also confirm that effective knowledge sharing may contribute to audit quality and efficiency (Duh et al., 2020). Therefore, this study proposes the following hypothesis:

*H5:* Knowledge sharing plays a moderating role between audit team’s EI and reduced audit quality behavior.

**Figure 1:**
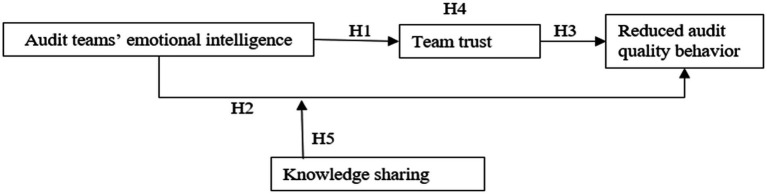
The theoretical model.

To summarize, this study constructed a model of the relationships between audit team’s EI, team trust, and reduced audit quality behavior. The theoretical model is depicted in Figure 1.

## Materials and methods

### Measurements of variables

To ensure that the reliability and validity met the requirements of the study, questionnaires measured each variable by drawing from existing common scales in the publicly available literature. Reliability and validity were pretested using data from 326 valid questionnaires. Apart from dependent variable and control variables, all variables were measured using a five-point Likert scale, i.e., score 1 = strongly disagree, 2 = disagree, 3 = neutral, 4 = agree, and 5 = strongly agree, which is shown in Table 1.

**Table 1:** Description of variables.

**Variable**	**Number**	**Measurement items**
Emotional intelligence (EI)	EI_1_	I have a good sense of why I have certain feelings most of the time.
	EI_2_	I always know my friends’ emotions from their behavior.
	EI_3_	I always set goals for myself and then try my best to achieve them.
	EI_4_	I am able to control my temper so that I can handle difficulties rationally.
Team trust (TT)	TT_1_	I consider my co-workers as people who can be trusted.
	TT_2_	I consider my co-workers as people who can be counted on to do what is right.
	TT_3_	I consider my co-workers as people who can be counted on to get the job done right.
	TT_4_	I consider my co-workers as people who are always faithful.
	TT_5_	I consider my co-workers as people who I have great confidence in.
Knowledge sharing (KS)	KS_1_	I share my job experience with my co-workers.
	KS_2_	I share my expertise at the request of my co-workers.
	KS_3_	I share my ideas about jobs with my co-workers.
	KS_4_	I talk about my tips on jobs with my co-workers.
Reduced audit quality behavior (RAQB)	RAQB_1_	Accepted weak client explanations.
	RAQB_2_	Failed to research an accounting principle.
	RAQB_3_	Made superficial reviews of documents.
	RAQB_4_	Prematurely signed-off on an audit step.
	RAQB_5_	Reduced work below what you considered reasonable.

#### Independent variable

The independent variable in this study was emotional intelligence (denoted by EI), which was measured using four dimensions of Wong and Law (2002). An example dimension is, “I have a good sense of why I have certain feelings most of the time.” This scale has been used in many studies conducted in the Chinese project context (Law et al., 2008).

#### Dependent variable

The dependent variable in this study was reduced audit quality behavior (denoted by RAQB). Using the instrument developed by from Smith and Emerson (2017), we measured reduced audit quality behavior using five items ranging from “never” to “nearly always,” such as “During the past year, how often have you acted in the following manner when carrying out an audit: accepted weak client explanations.”

#### Intermediary variable

The intermediary variable in this study was team trust (denoted by TT), which was assessed using scale items from work of Yilmaz and Hunt (2001). The scale is more in line with audit team’s situation. Team trust was assessed using five items, such as “I consider my co-workers as people who(m) can be trusted.”

#### Moderating variable

The moderating variable of this study was knowledge sharing (denoted by KS). Referring mainly to Lin (2007), we measured knowledge sharing by four interview questions. Participants were asked to evaluate their knowledge sharing behavior. Examples of these scale items are as follows: “I share my job experience with my co-workers” and “I share my professional knowledge at the request of co-workers.”

#### Control variables

We included a set of control variables following prior research on audit quality in the statistical analysis (Gul et al., 2013). We controlled the potential influence of the following variables: educational background (1 = Major in finance, accounting and auditing, 2 = other), experience in auditing (1 = less than or equal to 15 years, 2 = between 16 and 25 years, 3 = more than or equal to 26 years), gender (1 = male, 2 = female), rank (1 = partner, 2 = manager, 3 = other), and level of education (1 = college degree or below, 2 = undergraduate, 3 = Master’s degree or above).

### Description of the sample

Since the variables included in this study were not available from public information, we collected data using a large-scale audit firm questionnaire. The scope of this study was 426 audit firms of different sizes registered in 16 provincial-level administrative regions in China, and the specific respondents were CPAs practicing in the target firms. A total of 426 questionnaires were distributed and collected on site through the opportunity of business training conducted by the Chinese Institute of CPA, and through the distribution of questionnaires by the first author’s former colleagues in audit firms. The returned questionnaires were screened according to the principle of deleting the questionnaires that were not filled in, not selected, and those with less than 18 questions, in order to ensure the validity of the information in the recovered questionnaires, and therefore the analysis of the questionnaires no longer involves missing data. A final total of 326 usable questionnaires were obtained, with an overall efficiency rate of 76.53%. The reason why the questionnaire response rate is not high is that the survey objects cover a wide range of regions and there are many questionnaires excluded according to the screening conditions.

Table 2 shows the descriptive statistics of the sample. Among the total number of respondents, 65.95% were male, and 34.05% were female; over 43% of subjects majored in finance, accounting, and auditing; holders of a college degree or below accounted for 12.27%, holders of an undergraduate degree accounted for 76.69%, and holders of a Master’s degree or above accounted for 11.04%. Respondents with less than or equal to 15 years auditing experience accounted for 28.53%, those between 16 and 25 years of auditing experience accounted for 46.93%, and those with more than or equal to 26 years of auditing experience accounted for 24.54%. Respondents with partner rank accounted for 10.74%, those with manager rank accounted for 24.85%, and those with other rank accounted for 64.42%.

**Table 2:** Descriptive statistics for the sample.

Characteristic	Classification	Frequency	Percentage (%)	Characteristic	Classification	Frequency	Percentage (%)
Gender	Male	215	65.95	Education	College degree or below	40	12.27
	Female	111	34.05		Undergraduate	250	76.69
Educational background	Major in finance, accounting and auditing	141	43.25		Master’s degree or above	36	11.04
	Other	185	56.75	Rank	Partner	35	10.74
Experience in auditing	Less than or equal to 15 years	93	28.53		Manager	81	24.85
	Between 16 and 25 years	153	46.93		Other	210	64.42

### Testing for reliability and validity

The Cronbach’s α coefficients for audit team’s emotional intelligence, team trust, and reduced audit quality behavior scales were 0.874, 0.908, and 0.915, respectively. These results indicated that the items had good internal consistency and reliability.

An exploratory factor analysis was conducted using the Kaiser-Meyer-Olkin test and Bartlett’s test of sphericity. The Kaiser-Meyer-Olkin values for audit team’s EI, team trust, and reduced audit quality behavior were 0.835, 0.897, and 0.901, respectively, and passed Bartlett’s spherical test (*p* = 0.000 < 0.01), which indicated that the construct validity of the questionnaire was good.

We constructed a measurement model containing four latent variables and 18 observed variables. The parameters of the model were estimated and tested using the maximum likelihood method of the covariance structure model, and the fit indices of the hypothesized four-factor model were obtained as follows: $\chi^{2}$/df = 1.951 (<3), RMSEA = 0.054 (<0.08), GFI = 0.921 (>0.90), and CFI = 0.971 (>0.90). According to the criteria for a good model fit, the data were well-fitted, providing support for the distinctiveness of the four constructs in the study.

We compared the square root of the average variance extracted (AVE) value of the latent variable itself with the correlation coefficient between different latent variables to determine the discrimination validity, and found that the square root of AVE of each variable was greater than the correlation coefficient between the variable and other variables, indicating that the measurement scale had good discrimination validity. Variable AVE and correlation coefficient are shown in Table 3.

**Table 3:** Value of AVE variable and correlation coefficient of potential variable.

Variable	Ave	Correlation coefficient			
		RAQB	TT	KS	EI
RAQB	0.684	0.827			
TT	0.663	−0.347^**^	0.814		
KS	0.741	−0.339^**^	0.399^**^	0.861	
EI	0.636	−0.253^**^	0.381^**^	0.110^*^	0.797

^**^Significant at *p* < 0.01 (two-tailed); * Significant at *p* < 0.05 (two-tailed); The diagonal number is the square root of the variable AVE.

## Results

### Testing of hypotheses

Firstly, to verify the H1 proposed in Section “audit team’s EI and team trust,” the following regression models were constructed as follows:


(1) $$\mathrm{TT}=\beta_{0}+\beta_{1}\mathrm{EI}+\beta_{2}^{`}\mathrm{CONT}+\in$$

Where, CONT represents control variables (gender, educational background, experience in auditing, rank, and education);∈ denotes residuals. TT and EI are in keeping with how the variables are defined in Table 1. The regression outcomes of equation (1) in Model 2 showed that audit team’s EI was positively related to team trust (β_1_ = 0.386, *p* < 0.01). Therefore, the H1 was supported.

We then examined the mediating effect of team trust between audit team’s emotional intelligence and reduced audit quality behavior, drawing on the four conditions for establishing mediation proposed by Baron and Kenny (1986). Audit team’s EI was negatively related to reduced audit quality behavior (Model 3, β = −0.276, *p* < 0.01). Therefore, the H2 was supported.

Team trust was negatively related to reduced audit quality behavior (Model 4, β = −0.302, *p* < 0.01). Therefore, Hypothesis 3 was supported.

When team trust was added, the relationship between audit team’s emotional intelligence and reduced audit quality behavior was weaker, although still significant (Model 4, β = −0.159, *p* < 0.01), which suggests partial mediation. To further evaluate the mediating effect, we used the Mode 4 of PROCESS (Hayes, 2012) to test the indirect effect. When the 95% CI of the sample-based Bootstrap does not contain zero, the indirect effect of team trust is significant. After controlling for the control variables, results showed that the mediating effect of team trust on the relationship between audit team’s emotional intelligence and reduced audit quality behavior was −0.302 and the 95% CI of sample-based Bootstrap (5,000) was (−0.416, −0.188; excluded zero). Taken together, Hypothesis 4, team trust mediated the relationship between audit team’s emotional intelligence and reduced audit quality behavior, was thus supported.

**Figure 2:**
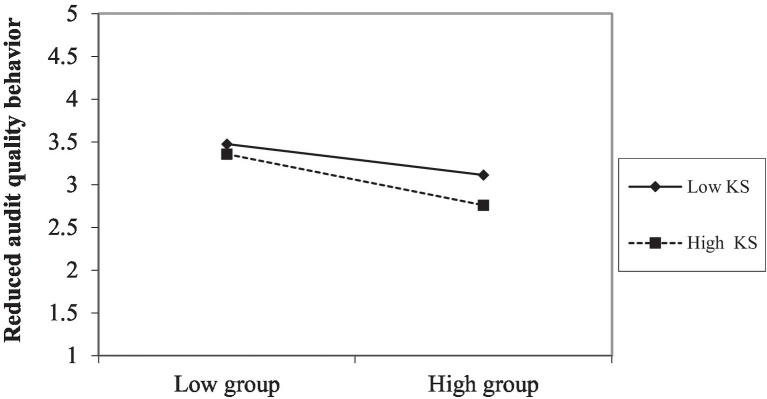
Interaction between audit team’s emotional intelligence and reduced audit quality behavior.

To further evaluate the moderating effect, we used the Mode 5 of PROCESS (Hayes, 2012) to test Hypothesis 5. H5 predicted that knowledge sharing moderated the relationship between audit team’s emotional intelligence and reduced audit quality behavior. After controlling for the control variables, the outcomes in Table 4 showed that the interaction between audit team’s emotional intelligence and knowledge sharing (EI*KS) is negatively related to reduced audit quality behavior (Model 5, β = −0.118, *p* < 0.01). To test for the existence of a moderating effect, it is inevitable that the contrast of such moderating effect is very sharp, i.e., the regression of the moderating effect is significant in the full sample of data, but this significance will only continue to exist in one of the subsamples, while this significance does not exist in the other subsample. Based on the above logic, we divided knowledge sharing into strong and weak subsample groups for the regression. Figure 2 showed that the negative relationship between audit team’s emotional intelligence and reduced audit quality behavior was significantly stronger, when knowledge sharing was at high group (β = −0.319, *p* < 0.01) than at low group (β = −0.043, ns), the difference is significant (Δ = −0.276, *p* < 0.01). Therefore, the H5 was supported.

**Table 4:** Results of relationship between audit team’s emotional intelligence and reduced audit quality behavior (*N* = 326).

Variable	Model 1	Model 2	Model 3	Model 4	Model 5
	RAQB	TT	RAQB	RAQB	RAQB
Constant	2.300^***^	2.022^***^	3.244^***^	3.854^***^	3.294^***^
Gender	0.08	0.096	0.06	0.089	0.021
Educational background	0.291^***^	−0.086	0.338^***^	0.312^***^	0.281^***^
Experience in auditing	0.055	−0.063	0.052	0.033	0.044
Rank	0.009	0.140^***^	0.016	0.058	0.044
Education	0.086	−0.131	0.036	−0.003	−0.067
EI		0.386^***^	−0.276^***^	−0.159^***^	−0.181^***^
TT				−0.302^***^	−0.197^***^
KS					−0.236^***^
EI^*^KS					−0.118^***^
*R* ^2^	0.018	0.16	0.085	0.397	0.467
*Adj-R* ^2^	0.002	0.145	0.068	0.157	0.218
*F*	1.16	18.964^***^	4.969^***^	8.472^***^	10.16^***^

^***^Significant at *p* < 0.01.

## Conclusion and discussion

### Conclusion

The goal of this study is to investigate whether, how, and when audit team’s EI influences reduced audit quality behavior. We introduce team trust and knowledge sharing as the mediator and moderator, respectively. Using a survey based on 326 respondents from Chinese audit firms, we confirm that audit team’s EI is negatively related to reduced audit quality behavior, and team trust negatively mediate the above relationship. In addition, knowledge sharing negatively moderates the relationship between audit team’s EI and reduced audit quality behavior. Specially, our findings suggest that audit firms’ knowledge sharing can reduce the behavior of absolute discretionary accruals and is positively related to the issuance of unfavorable audit opinions. Both of which indicate that when the knowledge sharing is higher, the audit quality reduction behavior can be reduced. The findings point to the importance of studying how audit team’s EI affects reduced audit quality behavior in audit firms, especially through the mediating role of team trust and moderating role of knowledge sharing.

### Theoretical implications

Our findings have several theoretical implications. Firstly, the research on reduced audit quality behavior has been mainly conducted from the cultivation of competence of auditors, audit independence, innovation and improvement of audit technology and methods, breach of psychological contract, improvement of audit standards and quality control system, etc. (Kusuma and Sukirman, 2017). This study actively explored the reduced audit quality behavior from the perspective of audit team’s EI in audit firms, constructed a theoretical model of audit team’s EI and reduced audit quality behavior, and obtained data through questionnaires, using structural equation model for empirical testing. Audit team’s EI can help improve audit quality by minimizing the tendency of auditors to participate in reduced audit quality behavior. This research outcome is consistent with that of Yang et al. (2018), who believe that EI can effectively reduce auditors’ dysfunctional behavioral tendencies and improve audit quality.

Secondly, over the past few decades, a large number of studies have found that positive behaviors, work contexts, and leadership types have a mediating effect between EI and work results (Kim et al., 2005; Cavazotte et al., 2012; Vidyarthi et al., 2014), and few studies have paid attention to the mediating effect of team trust. This study examined the audit team’s EI through the team trust variable in the causal chain to alleviate the audit quality reduction behavior.

Thirdly, the outcomes of this study established the mechanism of knowledge sharing in the audit team’s audit quality reduction behavior. At present, many researches regard EI as an important factor affecting audit quality, and find that EI is positively related to job satisfaction, work behavior, and work performance (Momm et al., 2014). Our findings are further based on this concept. It is found that the audit team with high knowledge sharing will have the audit quality reduction behavior only when its EI is high; In the audit team with low knowledge sharing, the audit team’s EI has no significant effect on reducing the audit quality behavior. Knowledge sharing, such as on-the-job training, experiences sharing in coping with difficult decisions, exchange of knowledge about new regulations and professional standards, and exchange of time-saving audit methods, will not only increase the audit team’s EI, but also improve the audit quality by converting knowledge into ability. Knowledge sharing can help audit firms leverage the skills, knowledge, and best practices of their professional staff (Vera-Munoz et al., 2006). Therefore, this study provides a new perspective and ideas for the audit quality management in audit firms, and expands and improves the research on audit quality.

### Managerial implications

Our findings have several practical implications. Firstly, this study provides guidance for human resource allocation and audit team building. EI is the lubricant to promote the harmonious and efficient work of the team, which can significantly reduce the audit quality reduction behavior. When more and more audit team members organize individuals from different regions, with different professional backgrounds, and at different ages to complete auditing together, EI is particularly important. In audit practice, managers or partners of audit firms need to understand the EI status of the audit team. In the absence of high EI talents, the use of a team organization system with a mix of high and low EI may help alleviate the plight of the lack of high EI talents. The audit firm’s managers can reduce and eliminate the adverse effects of reduced audit quality behavior through the emotional management of the audit team.

Secondly, this study promotes the audit firms to fully develop the value of EI of the audit team. EI is a potential resource that depends on individuals. This resource can play two roles, one is individual level, and the other is team level. For auditors, it is necessary to manage and control their EI during the audit process. For audit firms, it is also important to leverage the team trust channel and enhance the team’s EI to improve the audit quality. The organization can provide employees with mandatory regular EI training as a stress management technology, which will improve their work performance (Slaski and Cartwright, 2002).

Thirdly, knowledge sharing has a moderating effect on the relationship between audit team’s EI and reduced audit quality behavior. Therefore, audit firms’ managers should develop relevant policies and incentives in the usual management process to allow more knowledge to be shared within the audit firm, such as encouraging work suggestions and self-development so that employees can share their knowledge with others. Managers can motivate employees to share their knowledge with others by encouraging them to participate in goal setting and self-realization. In addition, managers can encourage audit teams to participate in decision-making so that employees have the opportunity to share knowledge with others. Through knowledge sharing, audit team members can enhance a greater sense of identification with their profession and become more willing to regulate their audit behavior in the process of implementing audit engagement (Knechel, 2013). The knowledge required to perform an audit may be unevenly distributed among audit firms or audit team members, and facilitating knowledge sharing can alleviate the audit quality reduction behavior.

### Limitations and future research

Three limitations of the present study should be mentioned. Firstly, this study only studied the impact of the audit team’s EI on reduced audit quality behavior. In the future, we can also explore whether and how the audit team’s time pressure, responsibility, leadership style, job satisfaction, task complexity, and other factors affect the audit team’s behavior of reducing audit quality. Secondly, this study only explored the mediating role of team trust and the moderating role of knowledge sharing. In the future, we can also explore whether there are other mediating variables and moderating variables that play a role between audit team’s EI and reduced audit quality behavior. Thirdly, due to the limitation of economic resources, the sample data we investigated only involved 426 audit firms, and only involved China. Therefore, there is still room for improvement in data collection, and the theoretical extrapolation validity needs to be further explored.

## Data availability statement

The raw data supporting the conclusions of this article will be made available by the authors, without undue reservation.

## Ethics statement

Ethical review and approval was not required for the study on human participants in accordance with the local legislation and institutional requirements. Written informed consent for participation was not required for this study in accordance with the national legislation and the institutional requirements.

## Author contributions

MZ contributed to establishment of the theory, the writing—original draft preparation, and the software. YL helped to analyze the data and editing. JL contributed to the calculations. All authors contributed to the article and approved the submitted version.

## Funding

This research was supported by the project of a core course for graduate students of Yunnan Normal University under grant no. YH2020-C09 and by the project for Case Database-Building of Yunnan Provincial Department of Education.

## Conflict of interest

The authors declare that the research was conducted in the absence of any commercial or financial relationships that could be construed as a potential conflict of interest.

## Publisher’s note

All claims expressed in this article are solely those of the authors and do not necessarily represent those of their affiliated organizations, or those of the publisher, the editors and the reviewers. Any product that may be evaluated in this article, or claim that may be made by its manufacturer, is not guaranteed or endorsed by the publisher.
